# Impact of insurance status on the survival of gallbladder cancer patients

**DOI:** 10.18632/oncotarget.18381

**Published:** 2017-06-06

**Authors:** Zhiqiang Chen, Wen Gao, Liyong Pu, Long Zhang, Guoyong Han, Qin Zhu, Xiangcheng Li, Jindao Wu, Xuehao Wang

**Affiliations:** ^1^ Department of Liver Surgery, The First Affiliated Hospital of Nanjing Medical University, Key Laboratory on Living Donor Liver Transplantation, National Health and Family Planning Commission, Nanjing, Jiangsu Province, China; ^2^ Department of Oncology, The First Affiliated Hospital of Nanjing Medical University, Nanjing, Jiangsu Province, China

**Keywords:** gallbladder cancer, insurance status, SEER, survival analysis

## Abstract

The prognostic significance of insurance status has been investigated in many types of malignancies, however, its impact on gallbladder cancer is yet not known. The purpose of this study was to determine the relationship between insurance status and gallbladder cancer survival. We searched the Surveillance, Epidemiology, and End Results dataset, and identified 1,729 gallbladder cancer cases. Kaplan–Meier methods and multivariable Cox regression models were used to analyze survival outcomes and risk factors. We found that individuals who had non-Medicaid insurance were more likely to be male, older, from wealthier area, and better-educated. Insurance status was confirmed as an independent prognostic factor for gallbladder cancer patients. Stratified analysis revealed that the uninsured status independently predicted unfavorable survival outcome at localized tumor stage and in white individuals. To conclude, insurance status is an important predictive factor for gallbladder cancer, and uninsured individuals are at the highest risk of death.

## INTRODUCTION

Gallbladder cancer (GBC) is the fifth most common gastrointestinal malignancy and the most frequent malignancy of the biliary tract, accounting for 80%-95% of biliary tree cancers around the world [1]. The etiology of this tumor is complex, and there is a strong association with cholelithiasis [2]. GBC is highly fatal and usually diagnosed at advanced stages due to absence of specific clinical findings in early stages [3]. It has been reported that the age-adjusted incidence rate of GBC is 1.4 per 100,000 in the United States, and is steadily increasing with age [4–6]. Despite recent advances in its diagnostic techniques and therapeutic managements, the prognostic outcome of patients with GBC remains dismal [7].

The correlation of insurance status with survival was demonstrated in different types of cancers. A higher risk of death associated with lack of health insurance or being a Medicaid beneficiary was found in younger patients with multiple myeloma [8]. Among patients with glioblastoma multiforme, uninsured status and Medicaid insurance indicated shorter survival compared to non-Medicaid insurance [9]. Survival was significantly better in privately insured patients with hepatocellular carcinoma [10]. In colorectal cancer patients, lack of insurance and Medicaid were independently associated with worse overall survival [11]. In obvious contrast, insurance status did not influence outcomes for adolescents and young adults with acute lymphoblastic leukemia [12]. The impact of insurance status on the survival of adult patients diagnosed with GBC, however, has not yet been examined. In the current study, we obtained data from the Surveillance, Epidemiology, and End Results (SEER) program, aiming to evaluate the association between insurance status and GBC cause-specific survival (GCSS) in the enrolled patients.

## RESULTS

### Patient population and characteristics

**Figure 1:**
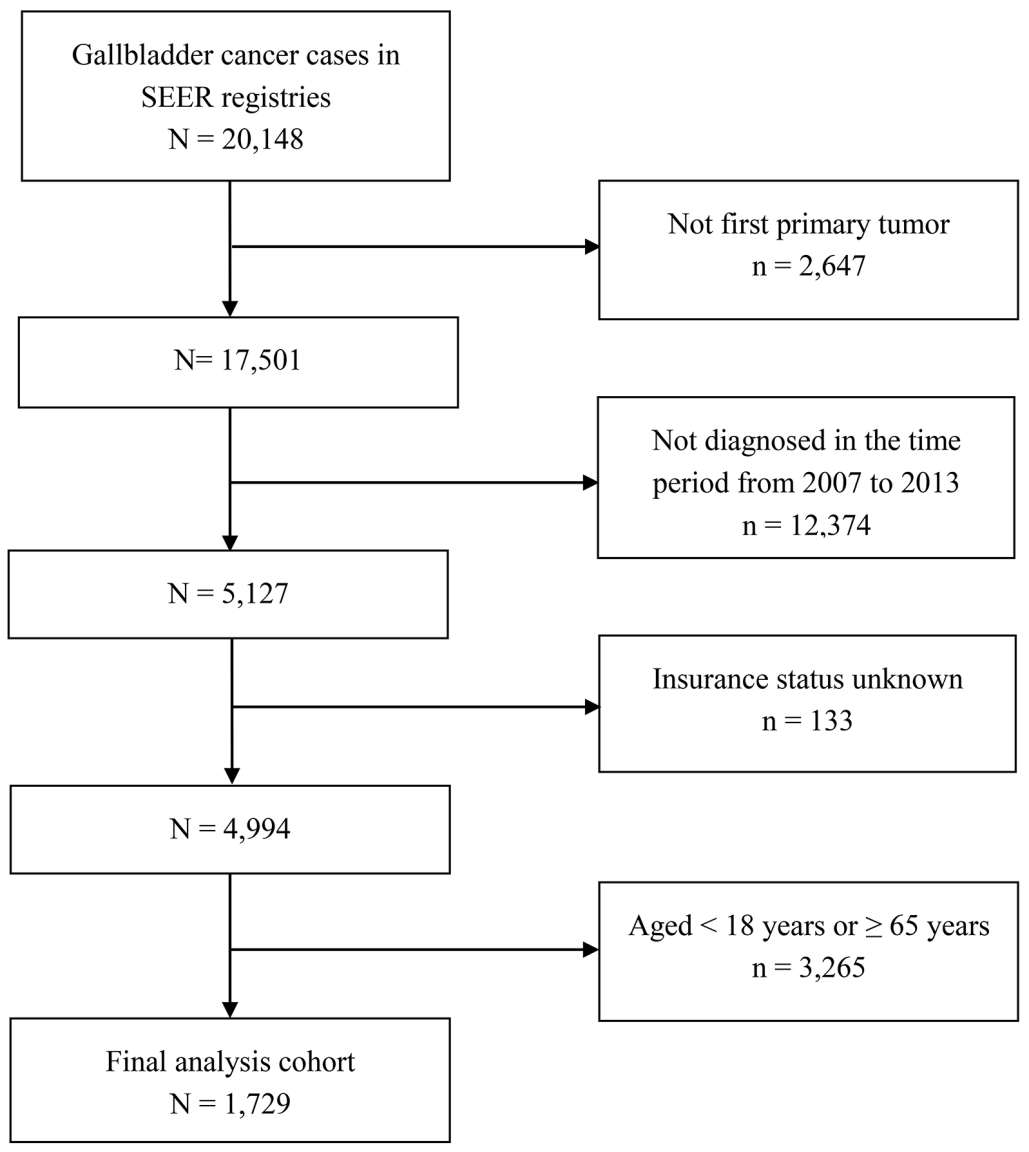
Flow diagram of patient selection for the current study

A total of 20,148 cases diagnosed with GBC were retrieved in the SEER database. After applying the inclusion and exclusion criteria, 1,729 GBC patients diagnosed during the 7-year study period (between 2007 and 2013) in the SEER were included in the final cohort. Figure 1 demonstrates the flow diagram for patient selection in the current study. Among the enrolled patients, 1,210 (70.0%) were females and 519 (30.0%) were males. A total of 1,217 patients (70.4%) were white, and 306 (17.7%) patients were black. The median age of included patients was 57 years. In the enrolled population, 1,160 patients (67.1%) had non-Medicaid insurance, 175 (10.1%) were uninsured, and 394 (22.8%) had Medicaid coverage. Significant differences were observed in subgroups including gender (*P*=0.001), age (*P*<0.001), pathological grading (*P*=0.005), county-level income (*P*<0.001), county-level education (*P*<0.001), and surgical therapy (*P*<0.001). Compared with the uninsured individuals, individuals who had non-Medicaid insurance were more likely to be male, older, from counties with higher income, and better-educated. In addition, patients with non-Medicaid insurance were more likely to receive surgical therapy. Table 1 illustrates variations in the distribution of patient demographics and tumor characteristics between different types of insurance coverage.

**Table 1:** Variations in insurance coverage in the enrolled population

Parameters	Total	Non-medicaid	Uninsured	Medicaid	*P*
	(n=1729)N(%)	(n=1160)N(%)	(n=175)N(%)	(n=394)N(%)	
Gender					0.001
Female	1210(70.0)	780(67.2)	139(79.4)	291(73.9)	
Male	519(30.0)	380(32.8)	36(20.6)	103(26.1)	
Age					<0.001
<57y	828(47.9)	510(44.0)	98(56.0)	220(55.8)	
≥57y	901(52.1)	650(56.0)	77(44.0)	174(44.2)	
Ethnicity					0.726
White	1217(70.4)	821(70.8)	122(69.7)	274(69.5)	
Black	306(17.7)	196(16.9)	35(20.0)	75(19.0)	
Other*	206(11.9)	143(12.3)	18(10.3)	45(11.4)	
Year of diagnosis					0.094
2007	244(14.1)	158(13.6)	30(17.1)	56(14.2)	
2008	190(11.0)	136(11.7)	18(10.3)	36(9.1)	
2009	260(15.0)	183(15.8)	29(16.6)	48(12.2)	
2010	228(13.2)	162(14.0)	21(12.0)	45(11.4)	
2011	263(15.2)	178(15.3)	28(16.0)	57(14.5)	
2012	272(15.7)	163(14.1)	26(14.9)	83(21.1)	
2013	272(15.7)	180(15.5)	23(13.1)	69(17.5)	
Histotype					0.087
Adenocarcinoma	1483(85.8)	1013(87.3)	146(83.4)	324(82.2)	
Squamous cell carcinoma	25(1.4)	13(1.1)	4(2.3)	8(2.0)	
Adenosquamous carcinoma	62(3.6)	43(3.7)	5(2.9)	14(3.6)	
Other^†^	159(9.2)	91(7.8)	20(11.4)	48(12.2)	
Pathological grading					0.005
Well/moderate	685(39.6)	481(41.5)	75(42.9)	129(32.7)	
Poor/anaplastic	538(31.1)	357(30.8)	41(23.4)	140(35.5)	
Unknown	506(29.3)	322(27.8)	59(33.7)	125(31.7)	
Tumor size					0.766
<3.5cm	458(26.5)	316(27.2)	43(24.6)	99(25.1)	
≥3.5cm	487(28.2)	328(28.3)	46(26.3)	113(28.7)	
Unknown	784(45.3)	516(44.5)	86(49.1)	182(46.2)	
TNM stage					0.265
I/II	868(50.2)	595(51.3)	90(51.4)	183(46.4)	
III/IV	780(45.1)	518(44.7)	75(42.9)	187(47.5)	
Unknown	81(4.7)	47(4.1)	10(5.7)	24(6.1)	
SEER stage					0.303
Localized	469(27.1)	327(28.2)	53(30.3)	89(22.6)	
Regional	370(21.4)	241(20.8)	36(20.6)	93(23.6)	
Distant	862(49.9)	576(49.7)	82(46.9)	204(51.8)	
Unstaged	28(1.6)	16(1.4)	4(2.3)	8(2.0)	
County-level income					<0.001
Quartile 1 (<US $59,290)	390(22.6)	252(21.7)	42(24.0)	96(24.4)	
Quartile 2 (US $59,290-$63,670)	465(26.9)	269(23.2)	52(29.7)	144(36.5)	
Quartile 3 (US $63,670-$81,810)	436(25.2)	309(26.6)	50(28.6)	77(19.5)	
Quartile 4 (≥US $81,810)	438(25.3)	330(28.4)	31(17.7)	77(19.5)	
County-level education					<0.001
Quartile 1 (<21.30%)	413(23.9)	265(22.8)	41(23.4)	107(27.2)	
Quartile 2 (21.30%-29.68%)	312(18.0)	241(20.8)	21(12.0)	50(12.7)	
Quartile 3 (29.68%-36.25%)	569(32.9)	334(28.8)	74(42.3)	161(40.9)	
Quartile 4 (≥36.25%)	435(25.2)	320(27.6)	39(22.3)	76(19.3)	
Surgical therapy					0.007
Yes	1152(66.6)	802(69.1)	109(62.3)	241(61.2)	
None/unknown	577(33.4)	358(30.9)	66(37.7)	153(38.8)	

SEER: Surveillance, Epidemiology, and End Results.

* Other includes American Indian/Alaska native, Asian/Pacific Islander, and unknown.

^†^ Other cancers include signet ring, small cell, giant and spindle cell, non-small cell carcinoma, carcinoma not otherwise specified, or undifferentiated carcinoma.

### Insurance status and GCSS

**Figure 2:**
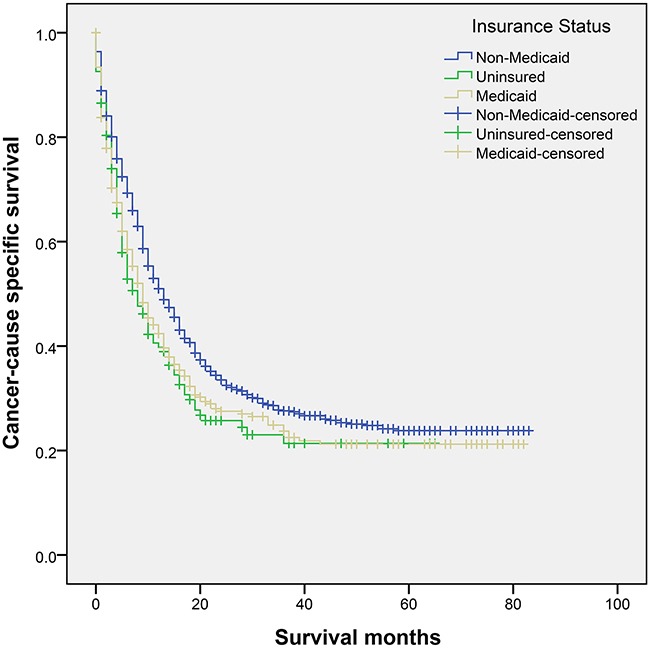
Survival curves in gallbladder cancer patients χ^2^=14.268,*P*=0.001.

The overall median survival of the included population was 9.0 months, with a 3-year GCSS of 12.0%. The 3-year GCSS was 27.6% in patients with non-Medicaid insurance, which was the highest compared with that in uninsured patients (21.4%) and in patients with Medicaid coverage (23.7%); all differences were significant according to the univariate log-rank test (*P*=0.001) (Figure 2). Gender (*P*=0.003), ethnicity (*P*=0.003), histotype (*P*<0.001), pathological grading (*P*<0.001), TNM stage (*P*<0.001), tumor size (*P*<0.001), SEER stage (*P*<0.001) and surgical therapy (*P*<0.001) were regarded as significant predictive factors for survival outcome by univariate analysis (Table 2). Multivariate analysis was carried out using the Cox proportional hazard model. The following nine factors were verified as independent prognostic factors for GBC (Table 2), including insurance status (uninsured, hazard ratio [HR] 1.279, 95% confidence interval [CI] 1.042-1.569), gender (male, HR 1.173, 95% CI 1.030-1.335), ethnicity (black, HR 1.227, 95% CI 1.053-1.430), histotype (squamous cell carcinoma, HR 1.884, 95% CI 1.213-2.925; adenosquamous carcinoma, HR 1.488, 95% CI 1.098-2.017), pathological grade (poor/anaplastic, HR 1.738, 95% CI 1.487-2.030), tumor size (≥3.5cm, HR 1.284, 95% CI 1.074-1.536), TNM stage (III/IV, HR 1.765, 95% CI 1.407-2.214), SEER stage (regional, HR 2.208, 95% CI 1.773-2.750; distant, HR 2.523, 95% CI 1.906-3.338), and surgical therapy (none/unknown, HR 1.813, 95% CI 1.533-2.143).

**Table 2:** Univariate and multivariate survival analysis for evaluating the influence of insurance status on gallbladder cancer cause-specific survival in SEER database

Variable	3-year CCS	Univariate analysis		Multivariate analysis	
		Log rank χ^2^ test	*P*	HR (95% CI)	*P*
Gender		8.694	0.003		0.016
Female	27.7%			Reference	
Male	22.2%			1.173(1.030-1.335)	
Age		1.949	0.163		NI
<57y	26.9%				
≥57y	25.3%				
Ethnicity		11.437	0.003		0.031
White	27.9%			Reference	
Black	18.2%			1.227(1.053-1.430)	0.009
Other*	27.1%			0.996(0.826-1.200)	0.962
Year of diagnosis		6.264	0.394		NI
2007	20.7%				
2008	27.5%				
2009	28.0%				
2010	25.1%				
2011	^††^				
2012	^††^				
2013	^††^				
Histotype		54.367	<0.001		0.003
Adenocarcinoma	28.6%			Reference	
Squamous cell carcinoma	5.7%			1.884(1.213-2.925)	0.005
Adenosquamous carcinoma	8.3%			1.488(1.098-2.017)	0.010
Other^†^	12.4%			1.171(0.965-1.421)	0.109
Pathological grading		237.074	<0.001		< 0.001
Well/moderate	45.0%			Reference	
Poor/anaplastic	15.6%			1.738(1.487-2.030)	< 0.001
Unknown	12.0%			1.122(0.929-1.355)	0.232
Tumor size		135.228	<0.001		< 0.001
<3.5cm	47.4%			Reference	
≥3.5cm	24.3%			1.284(1.074-1.536)	0.006
Unknown	15.0%			1.618(1.369-1.911)	< 0.001
TNM stage		485.792	<0.001		< 0.001
I/II	45.3%			Reference	
III/IV	4.6%			1.765(1.407-2.214)	< 0.001
Unknown	26.0%			1.541(1.058-2.245)	0.024
SEER stage		492.424	<0.001		< 0.001
Localized	64.1%			Reference	
Regional	25.0%			2.208(1.773-2.750)	< 0.001
Distant	6.5%			2.523(1.906-3.338)	< 0.001
Unstaged	18.6%			1.651(0.939-2.905)	0.082
County-level income		0.600	0.896		NI
Quartile 1 (<US $59,290)	25.8%				
Quartile 2 (US $59,290-$63,670)	28.1%				
Quartile 3 (US $63,670-$81,810)	26.3%				
Quartile 4 (≥US $81,810)	23.4%				
County-level education		2.693	0.441		NI
Quartile 1 (<21.30%)	30.5%				
Quartile 2 (21.30%-29.68%)	24.4%				
Quartile 3 (29.68%-36.25%)	25.5%				
Quartile 4 (≥36.25%)	23.6%				
Surgical therapy		459.917	< 0.001		< 0.001
Yes	37.4%			Reference	
None/unknown	2.9%			1.813(1.533-2.143)	
Insurance status		14.268	0.001		0.045
Non-medicaid	27.6%			Reference	
Uninsured	21.4%			1.279(1.042-1.569)	0.019
Medicaid	23.7%			1.109(0.959-1.282)	0.162

SEER: Surveillance, Epidemiology, and End Results; CCS: cancer cause-specific survival; HR: hazard ratio; CI: confidence interval; NI: not included in the multivariate survival analysis.

* Other includes American Indian/Alaska native, Asian/Pacific Islander, and unknown.

^†^ Other cancers include signet ring, small cell, giant and spindle cell, non-small cell carcinoma, carcinoma not otherwise specified, or undifferentiated carcinoma.

^††^ Because the follow-up records in SEER dataset ended in 2013, its 3-year CCS did not exist.

### Subgroup analysis of insurance status on GCSS based on SEER stage

**Figure 3:**
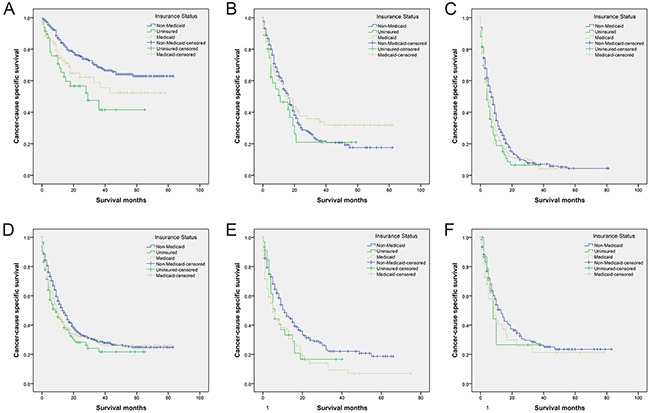
Survival curves in gallbladder cancer patients according to insurance status **(A)** SEER localized stage: χ^2^= 14.140 (*P*=0.001); **(B)** SEER regional stage: χ^2^= 2.139 (*P*=0.343); **(C)** SEER distant stage: χ^2^= 9.093 (*P*=0.011); **(D)** White: χ^2^= 6.540 (*P*=0.038); **(E)** Black: χ^2^= 10.508 (*P*=0.005); **(F)** American Indian/Alaska native, Asian/Pacific Islander: χ^2^= 0.922 (*P*=0.675).

As shown in Table 3 and Figure 3A-3C, we examined the effects of insurance status on GCSS at each SEER stage. Univariate analysis showed that patients with non-Medicaid insurance had the highest survival rate for both localized stage tumors and distant stage tumors. Individuals with non-Medicaid insurance had a 26.8% increase in 3-year GCSS compared with uninsured individuals (68.4% vs 41.6%, *P*<0.001), and a 9.5% increase compared with individuals with Medicaid coverage (68.4% vs 58.9%, *P*=0.020) for localized stage tumors. For distant stage tumors, non-Medicaid patients had a 0.6% increase in 3-year GCSS compared to uninsured patients (7.1% vs 6.5%, *P*=0.012), and a 1.6% increase compared to Medicaid recipients (7.1% vs 5.5%, *P*=0.031). The significant differences, however, were not observed in patients with regional stage tumors according to the results of univariate analysis (*P*=0.343). Multivariate Cox regression analyses were performed for different SEER stages. Insurance status was validated as an independent predictor of GBC survival at localized stage (uninsured, HR 2.122, 95% CI 1.297-3.473; Medicaid, HR 1.590, 95% CI 1.038-2.435). No significant results were found at SEER regional or distant stage in multivariate analyses.

**Table 3:** Univariate and multivariate survival analysis of insurance status on gallbladder cancer cause-specific survival based on different SEER stages

Variable	3-year CCS	Univariate analysis		Multivariate analysis	
		Log rank χ^2^ test	*P*	HR (95% CI)	*P*
**SEER stage**					
**Localized**					
**Insurance status**		14.140	0.001		0.006
Non-medicaid	68.4%	Reference		Reference	
Uninsured	41.6%	12.258	< 0.001	2.122(1.297-3.473)	0.003
Medicaid	58.9%	5.435	0.020	1.590(1.038-2.435)	0.033
**Regional**					
**Insurance Status**		2.139	0.343		NI
Non-Medicaid	21.8%	Reference			
Uninsured	20.9%	1.218	0.270		
Medicaid	34.0%	0.792	0.373		
**Distant**					
**Insurance Status**		9.093	0.011		NI
Non-Medicaid	7.1%	Reference			
Uninsured	6.5%	6.312	0.012		
Medicaid	5.5%	4.651	0.031		

SEER: Surveillance, Epidemiology, and End Results; CCS: cancer cause-specific survival; HR: hazard ratio; CI: confidence interval; NI: not included in the multivariate survival analysis.

### Subgroup analysis of insurance status on GCSS according to ethnicity

We further assessed the correlation of insurance status with cancer cause-specific survival according to different ethnicities (Table 4 and Figure 3D-3F). Compared to uninsured patients and Medicaid beneficiaries, patients with non-Medicaid insurance had the highest 3-year GCSS in all subgroups. Univariate analysis of insurance status revealed that non-Medicaid patients had a better 3-year GCSS compared to uninsured patients for white individuals (28.6% vs 21.7%, *P*=0.019). Multivariate analysis confirmed the independent prognostic effect of insurance status in white individuals (uninsured, HR 1.421, 95% CI 1.109-1.822). For black individuals, univariate analysis indicated that patients with non-Medicaid insurance had a better 3-year GCSS compared Medicaid beneficiaries (22.1% vs 9.3%, *P*=0.002). The influence of insurance status on GBC survival was not statistically significant in the subgroup of American Indian/Alaska native and Asian/Pacific Islander.

**Table 4:** Univariate and multivariate survival analysis of insurance status on gallbladder cancer cause-specific survival based on different ethnicities

Variable	3-year CCS	Univariate analysis		Multivariate analysis	
		Log rank χ^2^ test	*P*	HR (95% CI)	*P*
**Ethnicity**					
**White**					
**Insurance status**		6.540	0.038		0.028
Non-medicaid	28.6%	Reference		Reference	
Uninsured	21.7%	5.546	0.019	1. 421(1.109-1.822)	0.005
Medicaid	29.0%	2.328	0.127	1.040(0.870-1.244)	0.665
**Black**					
**Insurance Status**		10.508	0.005		NI
Non-Medicaid	22.1%	Reference			
Uninsured	16.6%	2.302	0.129		
Medicaid	9.3%	9.639	0.002		
**American Indian/Alaska native and Asian/Pacific Islander**					
**Insurance Status**		0.922	0.630		NI
Non-Medicaid	27.4%	Reference			
Uninsured	26.6%	0.176	0.675		
Medicaid	21.3%	0.864	0.353		

CCS: cancer cause-specific survival; HR: hazard ratio; CI: confidence interval; NI: not included in the multivariate survival analysis.

## DISCUSSION

GBC is a highly malignant cancer known for its aggressive biological nature and poor clinical presentation. Complete surgical resection is the only curative option available, but more than 90% of GBC patients are with un-resectable or metastatic disease [13]. Despite improved results of chemotherapy and surgery, the long-term outcome remains disappointing [14]. Thus, efforts are needed to identify factors contributing to prognosis of GBC. Previous studies have established several independent prognostic factors in patients with GBC. T stage, N stage, grade and histology are independent predictors of survival for gallbladder adenocarcinoma [15]. Tumor penetration of the gallbladder wall and pathologically confirmed lymph node involvement carry poor prognosis [16]. Studies in recent years have shown the importance of sociodemographic factors for survival in patients on GBC survival. It has been confirmed that marital status is an important prognostic risk factor for survival in patients with GBC treated with surgical resection [17]. To the best of our knowledge, our study is the first to associate insurance status with survival among patients diagnosed with GBC.

According to the results presented herein, patients with non-Medicaid insurance were more likely to be male, older, from richer area, and better-educated, which is in agreement with observations from previous studies that also utilized the SEER database [18, 19]. Non-Medicaid patients had the highest 3-year cancer-specific survival compared with uninsured patients and Medicaid recipients. Both patient- and tumor-related features may contribute to the heterogeneity of the study, and exert an effect on the prognosis of GBC patients. In the current study, we controlled for several variables that might lead to heterogeneity and attempted to demonstrate the association between insurance status and GBC survival. Cox proportional hazard analysis was performed, and the uninsured status was confirmed as an independent predictive factor of shorter survival in patients with GBC after adjusting for covariates including gender, ethnicity, histotype, pathological grading, tumor size, tumor stage, and surgical therapy. Stratified analysis of survival based on different SEER stages and ethnicities revealed that the uninsured status independently predicted unfavorable survival outcome at SEER localized stage and in white individuals. However, because of insufficient data, we did not further investigate other potential contributing factors such as genetic characteristics, comorbidities, operation methods, and hospital volume. Differences in the biological, psychological and social characteristics of the enrolled individuals may lead to the heterogeneity in the study, and potentially have an influence on the results. More large-scale studies are warranted to examine the associations and explore the underlying mechanisms.

One hypothesis for the survival differences between insured and uninsured patients is that insurance status may indirectly indicate the socioeconomic status of the individual. On one hand, it has been demonstrated that residence in counties with higher levels of poverty and rural residence were associated with being uninsured versus having non-Medicaid insurance [19]. Uninsured patients are less likely to schedule recommended surgery due to potential economic constraints. On the other hand, individuals with financial capacity and social support may have easier access to high-quality home and hospital care, which might lead to potential advantages in survival outcome [20]. Insured individuals are more likely to have regular access to health care [21]. An alternative explanation is that uninsured patients may experience medical comorbidities that potentially preclude surgical treatment, while insured patients may have lower levels of comorbidity. As SEER dataset did not provide detailed information about patient comorbidity, we could not further investigate this correlation.

Medicaid beneficiaries were described as underinsured or inadequately insured in other types of malignancies such as lymphoma, pediatric cancers and head and neck cancers [22, 23]. Interestingly, the results according to the multivariate analyses suggested that there were no significant differences between non-Medicaid patients and Medicaid beneficiaries in GBC (*P*=0.162). Nevertheless, uninsured patients had worse survival outcome compared to patients with insurance coverage (Non-Medicaid or Medicaid). Further studies with larger sample size are needed to verify this finding.

In spite of our efforts to make a comprehensive and accurate analysis, there are several limitations to this study. First, the retrospective nature of this study may lead to bias and potentially have an influence on the results. Second, it has been widely acknowledged that the operation methods and comorbidities have an impact on the prognosis of cancer patients. As the variables provided in SEER database were limited, we could not adjust the results for these covariates. Third, information on the duration of insurance was not provided in SEER dataset. As a result, we could not distinguish between those who had Medicaid coverage for many years and those enrolled at the time of diagnosis. Fourth, the insurance information for those aged 65 years or older is currently not clearly recorded in SEER database, therefore we excluded this age population. Fifth, income and education status at individual level were unobtainable from SEER dataset, and both of these variates might result in treatment decisions. Finally, it is noteworthy that this study was limited as the results shown can only demonstrate the correlation in specific SEER regions and should be interpreted with cautions while being applied in other regions. The under-registration and misclassification within and among counties might also result in bias.

In conclusion, we found that insurance status was an independent predictor for survival in patients with GBC. Uninsured individuals were at the highest risk compared to non-Medicaid patients and Medicaid recipients. Subgroup analysis suggested the uninsured status independently predicted unfavorable survival outcome at localized stage and in white individuals with GBC. Future studies are needed to validate these findings and investigate the underlying mechanisms of survival disadvantage in uninsured patients.

## MATERIALS AND METHODS

### Patient selection in the SEER database

All primary data were extracted from the SEER database using SEER*Stat version 8.3.2. The SEER includes population-based cancer populations reported in the Alaska, California, Connecticut, Georgia, Hawaii, Iowa, Kentucky, Louisiana, Michigan, New Jersey, New Mexico, Utah, and Washington registries, representing approximately 28% of the population in the United States. The SEER data have been widely used for studies investigating the relationship between insurance status and tumor characteristics [24–26].

GBCs were identified by the topography code C23.9 for gallbladder with the following International Classification of Diseases for Oncology, 3^rd^ Edition (ICD-O-3) codes as previously reported [17]: adenocarcinoma (8140, 8141, 8143 and 8147), mucinous adenocarcinoma (8480 and 8481), papillary adenocarcinoma (8260-8263), adenocarcinoma with metaplasia (8571-8576), duct carcinoma (8500, 8501, 8503, 8504, 8507 and 8508), papillary carcinoma (8050-8052), squamous cell carcinoma (8070-8076 and 8078), adenosquamous carcinoma (8560 and 8562), or other cancers including signet ring (8490), small cell (8041 and 8043), giant and spindle cell (8030–8035), non-small cell carcinoma (8046), carcinoma not otherwise specified (8010-8015) or undifferentiated carcinoma (8020-8022).

Inclusion criteria were as follows: (1) patients with GBC as their primary diagnosis; (2) patients diagnosed with GBC in the time period from January 1^st^, 2007 to December 31^st^, 2013, considering that the SEER program began collecting insurance status in 2007. The exclusion criteria for patients included the following: (1) patients with unobtainable insurance information were excluded; (2) patients aged < 18 years were excluded; (3) patients aged 65 years or older were excluded as it was the age that most patients are eligible for Medicare, which is currently not clearly coded for individuals in SEER program and not recommended to be used in this age population.

GCSS was the primary focus of this study, and was calculated from the date of diagnosis of GBC and the date of GBC cause-specific death. Deaths attributed to GBC were treated as events, and deaths from other causes were treated as censored observations.

### Patient demographics and clinicopathological variables

Potentially relevant patient and clinicopathological variables were included in the analyses. Insurance status was defined as non-Medicaid (including non-Medicaid and no specifics), uninsured, and Medicaid (any Medicaid). Tumor size was categorized into two groups: <3.5cm and ≥3.5cm. The selected cutoff value of 3.5cm represented the median size of all GBC. The TNM stage was established according to the criteria described in the American Joint Committee on Cancer staging atlas (the 6^th^ edition). According to the SEER staging system, diseases that confined to the organ of origin were defined as localized, diseases that invaded locally or metastasized to regional lymph nodes were considered to be regional, and diseases that spread to remote organs were regarded as distant. Household income and level of education could not be obtained in SEER as individual-level data, and therefore we used county-level data. Median household income within the county of residence at the time of diagnosis was chosen to represent the county-level income level at the time of diagnosis, and percentage of adult individuals with at least a bachelor's degree was selected to represent the county-level education level.

### Statistical analysis

Differences in baseline parameters were analyzed by chi-squared (χ^2^) test for categorical variables. Survival curves were generated using the Kaplan-Meier estimates, and log-rank χ^2^ tests were performed to compare differences between subgroups of each variable. Multivariate Cox proportional hazard models were built to determine risk factors for survival outcomes. Results were considered statistically significant when a two-sided *P* value less than 0.05 was achieved. All statistical analyses were conducted using SPSS software (version 21.0; Statistics Package for Social Science, Chicago, IL).

## Abbreviations

GBC: gallbladder cancer
SEER: Surveillance, Epidemiology, and End Results
GCSS: gallbladder cancer cause-specific survival
HR: hazard ratio
CI: confidence interval
ICD-O-3: International Classification of Diseases for Oncology, 3rd Edition
